# The association between aberrant salience and psychotic experiences in general population twins, and genetic vulnerability as a modifier

**DOI:** 10.1186/s12888-024-06176-2

**Published:** 2024-10-26

**Authors:** Marjan Drukker, Tatvan Todor, Jelle Bongaarts, Eleonora Broggi, Mihika Kelkar, Thomas Wigglesworth, Kayle Verhiel, Karel van Leeuwen, Meinte Koster, Catherine Derom, Evert Thiery, Marc De Hert, Claudia Menne-Lothmann, Jeroen Decoster, Dina Collip, Ruud van Winkel, Nele Jacobs, Sinan Guloksuz, Bart Rutten, Jim van Os

**Affiliations:** 1 Department of Psychiatry and Neuropsychology, School of Mental Health and Neuroscience (MHeNS), Maastricht University Medical Centre, Maastricht, the Netherlands; 2 Faculty of Health, Medicine and Life Sciences, Maastricht University, Maastricht, The Netherlands; 3 Department of Obstetrics and Gynecology, Ghent University Hospitals, Ghent University, Ghent, Belgium; 4 Department of Neurology, Ghent University Hospital, Ghent University, Ghent, Belgium; 5 University Psychiatric Centre, Katholieke Universiteit Leuven, Kortenberg, Belgium; 6 Department of Neurosciences, Centre for Clinical Psychiatry, Katholieke Universiteit Leuven, Louvain, Belgium; 7 Leuven Brain Institute, Katholieke Universiteit Leuven, Louvain, Belgium; 8 Antwerp Health, Law and Ethic Chair, University of Antwerp, Antwerp, Belgium; 9 Psychiatric Care Sint-Kamillus, Brothers of Charity, Bierbeek, Belgium; 10 Department of Neurosciences, Research Group Psychiatry, Center for Clinical Psychiatry, Katholieke Universiteit Leuven, Louvain, Belgium; 11 University Psychiatric Center, Katholieke Universiteit Leuven, Leuven, Belgium; 12 Faculty of Psychology, Open Universiteit, Heerlen, The Netherlands; 13 Department of Psychiatry, Yale University School of Medicine, New Haven, CT USA; 14 Department of Psychiatry, Brain Centre Rudolf Magnus, University Medical Centre Utrecht, Utrecht, The Netherlands; 15 Department of Psychosis Studies, Institute of Psychiatry, King’s College London, King’s Health Partners, London, UK

**Keywords:** Aberrant salience, Genetic vulnerability, Subclinical psychotic symptoms

## Abstract

**Background:**

Previous studies assessing the hypothesis that the construct of ‘aberrant salience’ is associated with psychosis and psychotic symptoms showed conflicting results. For this reason, the association between measures to index aberrant salience and subclinical psychotic symptoms in a general population sample was analysed. In addition, genetic vulnerability was added to the analysis as a modifier to test the hypothesis that modification by genetic vulnerability may explain variability in the results.

**Methods:**

The TwinssCan project obtained data from general population twins (N = 887). CAPE (Community Assessment of Psychic Experience) scores were used to index psychotic experiences. Aberrant salience was assessed with white noise task and ambiguous situations task.

**Results:**

Measures of aberrant salience were not associated with psychotic experiences, nor was there evidence for an interaction with genetic predisposition in this association (Z = 1.08, p = 0.282).

**Conclusions:**

Various studies including the present could not replicate the association between aberrant salience and psychotic experiences in general population samples. The conflicting findings might be explained by moderation by genetic vulnerability, but results are inconsistent. If there was evidence for a main effect or interaction, this was in the positive symptom scale only. On the other hand, the association was more robust in so-called ‘ultra-high risk’ patients and first episode psychosis patients. Thus, this association may represent a state-dependent association, present only at the more severe end of the psychosis spectrum.

## Introduction

Psychotic disorders are characterised by altered perception of, and meaning associated with, the environment [1], expressed in the form of hallucinations and delusions that constitute the positive symptoms of psychotic disorders. These symptoms may be rooted in normal mentation, in the sense that human perception is characterised by a highly personal and intricate, affectively driven process of allocating salience to ‘bottom-up’ sensory information. This may vary within and between persons in ways that can easily give rise to misunderstandings, deviation from ‘shared reality’ and experiences that resemble psychotic symptoms, but below the threshold of clinical relevance, so-called psychotic experiences. Psychosis is expressed across a spectrum of severity, ranging from subclinical psychotic symptoms to chronic psychotic illness. It is suspected that variation in the processes of perception and meaning across the spectrum of psychosis severity is multifactorial, affected by subtle neurodevelopmental alterations, environmental stressors, and genetic variation [2, 3].

### Aberrant salience and psychosis

It has been postulated that underlying alterations, hypothesised at the level of upregulation of presynaptic dopamine regulation (see below), may disrupt the process of contextually driven salience, resulting in aberrant allocation of salience to stimuli [4]. Evidence supporting this hypothesis is derived from study populations in the prodromal phase of psychosis spectrum disorder [5, 6]. When salience is attributed to otherwise meaningless internal stimuli, these stimuli may appear to be externally generated, resulting in, for example, verbal and visual hallucinatory phenomena [7, 8]*.* However, evidence is inconsistent. Both in first episode psychosis patients and in individuals meeting variably defined criteria for ‘ultra-high risk’ or ‘clinical high risk’, aberrant salience is higher than in healthy controls, and thus theoretically could be used as a possible marker for psychosis spectrum disorder [9–12]. In addition, two previous studies showed an association between aberrant salience and psychotic experiences below the clinical threshold [13, 14]. However, two further studies with similar designs did not show an association with psychotic experiences [15, 16].

Several tests indexing aberrant salience are available, including the white noise task and the ambiguous situations task [16, 17]. The white noise task assesses the attribution of meaning to otherwise meaningless, external auditory stimuli [16]. Individuals are presented with white noise and asked if they heard spoken speech in the white noise, and to what degree this speech was affectively positive or negative. The ambiguous situations task investigates the presence of interpretation bias when presented with an ambiguous event (see methods for a more detailed explanation) [17].

### Hypothesized mechanism: dopamine dysregulation

It has been suggested that structural brain abnormalities in the prefrontal cortex and aberrant glutamatergic activity in early development may represent an underlying factor impacting subcortical disinhibition observed later in adolescence [3]. Alterations in the capacity to inhibit subcortical areas is hypothesized to result in inaccurate processing of stimuli. The brain may try to compensate for this inaccuracy by amplifying dopamine signalling. As dopamine may be involved in the process of regulating attribution of meaning to a stimulus, defined as salience, it has been stated that hyperactivity of the system may lead to a shift from adaptive to aberrant salience, for example in individuals deemed at ultra-high risk or with a diagnosis of psychotic disorder [3, 7].

Environmental stressors, such as childhood adversity, urbanisation and immigration may similarly affect dopamine signalling. Thus, there is evidence that stress can sensitize the dopamine system, which can trigger an exacerbated dopamine response as hypothesized to occur in patients with psychotic disorder [3, 4]. Heightened dopamine responses, in turn, may give rise to the attribution of aberrant salience to stimuli. The eventual occurrence of psychotic symptoms may further increase stress, forming a loop reaction [3, 4].

### Genetic vulnerability

**Fig. 1:**
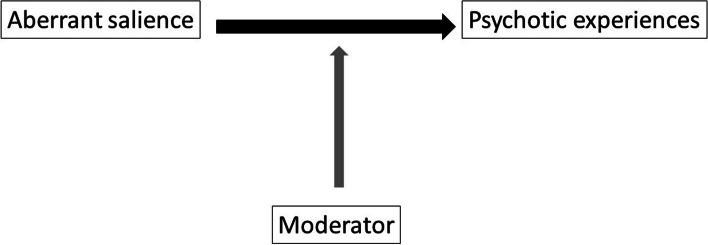
Hypothesis for moderation in the association between aberrant salience and psychotic symptoms

It is likely that genetic predisposition is not only directly associated with psychotic symptoms, but also modifies the strength of other risk factors in the sense of rendering the person more or less sensitive to the psychotogenic effects of the environment (Fig. 1) [18]. As the experience of (aberrant) salience is environmentally mediated, genetic factors likely will explain differences between individuals in the intensity of the aberrant salience response to environmental variation and the risk that it may progress to psychotic symptoms. A wide variety of genetic loci has been associated with psychosis [19]. Given that a great number of alleles are involved in the development of psychosis, a polygenic risk score for schizophrenia (PRS) has been proposed [20]. The PRS combines the risk of a wide variety of genetic variants (e.g. single nucleotide polymorphisms) into a single summary score as a proxy for genetic vulnerability of a certain phenotype [19, 20].

#### Genetic vulnerability and positive symptoms

Genetic research provides further insight into the mechanisms underlying dopamine dysregulation and its role in psychosis. Certain genetic variations correlate strongly with measures of subclinical psychotic experiences [21]. Positive symptoms in psychosis, such as hallucinations (hearing or seeing things that are not present) and delusions (firmly held false beliefs), have been associated with genetic factors [22, 23]. These symptoms represent an excess or distortion of normal functions and are direct manifestations of aberrant salience. Evidence suggests that attempts to link genetic factors to expression of positive symptoms may be more straightforward (and perhaps more productive) because of the higher specificity of positive symptoms compared to other types of symptomologies in psychosis, such as negative and depressive symptoms [23]. Thus, the association between PRS and psychotic symptoms may be specifically found in positive symptoms [22]. If we can replicate findings of selective genetic sensitivity to positive symptoms, it is tempting to argue that this may reflect a crucial aspect of how genetic factors may amplify the effects of environmental stressors on dopamine pathways, thereby increasing the risk of developing psychosis. This genetic vulnerability, coupled with the previously discussed environmental factors, helps illustrate the complex interplay of genetics and environmental influences in the manifestation of psychotic disorders.

### Aim

Due to conflicting results in previous studies [13–16], we reanalysed longitudinal data from the TwinssCan Project [16]. While in the earlier study only baseline data were analysed, we now were able to also include follow-up data. First, we investigated the association between aberrant salience and subclinical psychotic symptoms. Both the psychotic symptoms sum score and the positive symptoms subscale were analysed. As opposed to above mentioned studies on aberrant salience and psychotic experiences in the general population [13, 15, 16], aberrant salience was analysed using two different assessments: the white noise task and the ambiguous situations task. Second, in order to understand the role of genetic variation, the paper aimed to analyse the influence of genetic background on the association between aberrant salience and the occurrence of psychotic experiences (as depicted in Fig. 1). Again, both the psychotic symptoms sum score and positive symptoms subscale were analysed.

## Methods

### Participants

The data utilised in the present study were sourced from the TwinssCan Project, which was co-funded and part of the European Network of National Networks Studying Gene-Environment Interactions in Schizophrenia [16, 24]. The primary aim of TwinssCan is to explore both the risk and protective factors for psychopathology development, as well as the contribution of genetic and nongenetic factors to intermediate phenotypes [25]. The participants were recruited from the East Flanders Prospective Twin Survey (EFPTS) ADDIN EN.CITE [16, 24]. All twins aged between 15 and 18 years registered in the Belgian multi-birth registry, the EFPTS, received a personal invitation letter. All other twins were invited in the newsletter that all twins registered in EFPTS received. This strategy was chosen to oversample adolescents [24]. There were no other in- and exclusion criteria.

Approval was obtained from the local ethics committee (Commissie Medische Ethiek van de Universitaire ziekenhuisen KU Leuven, Nr. B32220107766). All methods were performed in accordance with the relevant guidelines and regulations.

The participants *(n* = *792*) were monozygotic or dizygotic twin pairs, triplets and their siblings, all aged between 15 and 35 years (mean = 17.47 years, 60% female). Baseline data (wave 1) were collected between April 2010 and April 2014 [25] and wave 2 data were collected one year later. Earlier, our first research question was assessed using baseline data only [16]. The present analyses are performed with baseline and follow-up data to increase power. All participants provided written informed consent, parents of participants younger than 18 years also provided informed consent [24].

### Instruments

#### CAPE for measuring psychotic experiences

The Community Assessment of Psychic Experiences (CAPE) is a self-report questionnaire consisting of 42 items (20 items on positive psychotic experiences, 14 items on negative psychotic experiences, and 8 items on depressive feelings) [26, 27]. Both the frequency (never, sometimes, often, nearly always) and the distress (not distressed, a bit distressed, quite distressed, very distressed) associated with these experiences are measured by the CAPE. The CAPE provides both an overall frequency sum score and a frequency sum score per dimension, and it provides a distress sum score [27]. For the present analyses, both the CAPE overall frequency sum score and the frequency sum score for the positive dimension were used.

#### Ambiguous situation task for measuring aberrant salience

The ambiguous situations task was the first tool used to measure aberrant salience [17]. During this test, the TwinssCan subjects were presented 10 texts on hypothetical situations. Subsequently, they were asked to fill in the last word of the text (no right or wrong) and to answer a comprehension question. Both providing the last word and answering the comprehension question ensure that the participant reads the text. First, all ten hypothetical situations were filled in. Subsequently, per hypothetical situation, four phrases were provided. Phrases were similar to the original text, but one had a negative interpretation (NT = negative target), and one had a positive interpretation (PT = positive target) of the original ambiguous situation. Subjects were asked how similar those phrases were to the original hypothetical situation (1-not similar at all – 4-very similar). The more similar the NT and PT phrases were rated, the more negative and positive interpretation bias there was. Per subject, sum scores for NT and PT were calculated. In addition, a sum score including both NT and PT (NTPT) was also used in the analysis. A higher target score was associated with a higher level of aberrant salience. Next to NT and PT, phrases could also be negative foil (NF) or positive foil (PF). A foil is a phrase that is similar, but not the same as the original ambiguous situation. These foils were added to make the task less transparent and were not used for analysis.

#### White noise task for measuring aberrant salience

The second tool to assess aberrant salience was the white noise task [13]. This is an auditory test in which subjects wear earphones and are presented different sound signals. Each participant receives 25 sound signals that can be divided into three conditions: (1) white noise, (2) white noise with clear audible neutral speech, (3) and white noise with hardly audible neutral speech. The sound signals were presented in random order over a time period of approximately 15 min. Participants were instructed to press one of the five buttons that associate the sound fragment with one of the following meanings: (1) positive speech, (2) negative speech, (3) neutral speech, (4) no speech heard, and (5) uncertain [13]. The variable of interest was the occurrence of hearing speech illusions in the white noise only condition, acknowledged by the fragment being linked to button 1, 2 or 3 by the subject. To determine whether speech illusions have generally been experienced during the white noise fragment, a composite variable called 'any speech illusion' was created that included the positive, negative, and neutral speech illusions. Any speech illusion heard in the white noise condition is interpreted as allocating meaning to normally meaningless signal, in line with the notion of aberrant salience.

#### Polygenic risk score for schizophrenia

For details of genotyping and calculation of PRS in two previous data collections, we refer to recent papers detailing these procedures [18, 28, 29]. We used the recent genome-wide association study (GWAS) of the Psychiatric Genomics Consortium of schizophrenia for PRS calculations. PRS-schizophrenia was created, using the same genotyping platform as for the earlier data collections, from best-estimate genotypes at six different *p-*thresholds (0.5, 0.1, 0.05, 5·10^–3^, 5·10^–5^, 5·10^–8^). For our primary analyses, we used the *p-*threshold of < 0.05, as this threshold explained most variation in the phenotype in the Psychiatric Genomics Consortium analysis [29].

### Statistical analysis

Data had a multilevel structure because multiple assessments (wave 1 and wave 2) were clustered in twins and twins were clustered in twin pairs. Mixed-effect linear regression analyses were performed in which CAPE-score was the dependent variable. Aberrant salience was the independent variable which was represented in the analysis by the white noise task and the ambiguous situations task. Any speech illusion from the white noise task and PT, NT and NTPT form the ambiguous situation test were used for analysis. For the second aim, aberrant salience x PRS interactions terms were added to the multilevel mixed-effects linear regression model. In all models age, gender, and parental education were a priori included in the model as confounding factors. Parental education was selected as a proxy for socioeconomic status [30–33] because the twins were relatively young and thus did not all finish their education. When PRS was included in the interaction term, analyses were adjusted for the first two principal components obtained with the PRS to control for population stratification (pc1 and pc2). All analyses were performed using STATA version 13 [34].

## Results

Because of the invitation procedure, respondents were relatively young (mean 17.5 years), but range was broad (15–34 years, Table 1). Forty percent were female and 72% had high-educated parents (bachelor or master degree).

**Table 1:** Descriptives of independent and dependent variables

**Section A: Continuous variables**				
	**Mean**	**SD**	**Range**	**Number of subjects (assessments)**
**CAPE (frequency)**^a^				
Cape total	64.6	10.1^a^	43–127	815 (1300)
Cape positive subscale	27.4	5.1^a^	20–62	815 (1300)
**Ambiguous situations**				
NT	22.9	4.5	10–40	828
PT	23.6	4.1	10–39	828
NTPT	46.6	5.9	20–78	828
**Age at wave 1 (years)**	17.5	3.68	15–34	688
**Section B: Categorical variables**				
	**Frequency**		**Percentage**	
**Sex**				
Male	338		40.3%	
Female	501		59.7%	
**Parental education (Socioeconomic status)**				
High	598		71.8%	
Medium	164		19.7%	
Low	71		8.5%	
**White noise**				
Any speech illusions	64		8.1%	

*CAPE *Community Assessment of Psychic Experience*, SD *Standard deviation

^a﻿^CAPE is assessed at baseline and at 1-year follow-up. Descriptives are multilevel and SD is SD between (total number of assessments is 1300, not all subjects provided CAPE twice)

### Association between aberrant salience and CAPE sum scores

Table 1 presents means, standard deviations, ranges, and frequencies of the CAPE, the ambiguous situations task (NT, PT, NTPT) and the white noise task, respectively. Table 2 presents regression coefficients of the associations between aberrant salience measures (ambiguous situation task, white noise task) and CAPE. Associations between ambiguous situations (NT, PT, NTPT) and CAPE total score and positive symptoms sub scale were not statistically significant. The association between the white noise task and the CAPE total score and positive symptoms was also not statistically significant.

**Table 2:** The association between aberrant salience and psychotic symptoms (CAPE)

	Psychotic symptoms, total		Positive symptoms subscale	
	B (95% CI)	*p-*value	B (95% CI)	*p-*value
**Ambiguous situations**				
NT	0.04 (-0.12; 0.20)	0.65	0.0 (-0.07; 0.09)	0.81
PT	0.10 (-0.08; 0.27)	0.27	0.06 (-0.02; 0.15)	0.14
NTPT	0.07 (-0.05; 0.20)	0.25	0.04 (-0.02; 0.10)	0.21
**White noise**				
Any speech illusions	-0.72 (-3.26; 1.81)	0.58	0.73 (0.53; 1.99)	0.26

Multilevel regression analysis of the association between independent variables white noise task and ambiguous situations and dependent variable CAPE total score controlled for gender, parental education, and age

*CAPE *Community Assessment of Psychic Experience

### Interaction between aberrant salience and PRS

When analysing the CAPE total score, only the interaction between PRS and NT was statistically significant (Table 3). However, when analysing CAPE positive symptoms, the interaction with PT and NT as well as white noise was statistically significant. When PRS was higher the association between the independent variables assessing aberrant salience and positive symptoms was stronger.

**Table 3:** Interactions PRS X Ambiguous situations or PRS X speech illusions when analysing psychotic symptoms (CAPE)

	Psychotic symptoms, total		Positive symptoms subscale	
	*Z* score (S.E.)	*p-*value	*Z* score (95% CI)	*p-*value
**Ambiguous situations and PRS**				
NT	**0.17 (0.08)**	**.03**	**0.14 (0.04)**	** < 0.01**
PT	-0.15 (0.09)	.09	**-0.1 (0.04)**	**.02**
NTPT	.05 (0.06)	.45	.05 (0.13)	.13
**White noise and PRS**				
Any speech illusions	1.32 (1.24)	.29	**1.28 (0.62)**	**.04**

Multilevel regression analysis of the interaction between independent variables (white noise task and ambiguous situations) and PRS when analysing CAPE total score and CAPE positive symptoms controlled for gender, parental education, and age

*CAPE *Community Assessment of Psychic Experience*, S.E. *Standard error

## Discussion

In the present study, aberrant salience was not associated with subclinical psychotic symptoms or positive symptoms, but there was some evidence that genetic variation was a moderator. Only one of four subgroups showed evidence for interaction between genetic vulnerability and aberrant salience (PRS X NT) when assessing psychotic symptoms sum score. However, when assessing positive symptoms 3 out of 4 measures of aberrant salience showed this interaction.

### Strengths and limitations

Strength of the present analysis is the power; multiple assessments per person were analysed using multilevel analysis (mixed). However, this also implied that despite the longitudinal character of the study, data were analysed cross-sectionally. Multiple observations per person were used to increase power rather than to assess associations over time. Power is the main reason why we reanalysed the data set used by Pries and colleagues [16]. Previous research was also cross-sectional. If cross-sectional analyses show no evidence for an association, it may not be productive to design a longitudinal study. A second methodological issue is that the study population is relatively young as adolescents between 15 and 18 years were oversampled. Thus, the study population is not representative for adult samples. However, results are similar as in research in general population adults (see below).

Furthermore, the present paper assessed one modifier only, i.e. genetic vulnerability. However, other modifiers are also hypothesized to play a role, but are not included in the present analysis. Previous research [35] showed that childhood trauma and life events were also no moderators in the association between white noise and psychotic experiences.

Finally, we performed a sensitivity analysis, in which PRS was replaced by cross-twin cross-trait genetic vulnerability [36]. Here monozygotic twins with a twin scoring high on a psychopathology scale are in the high risk category and dizygotic twins with a twin scoring high on this psychopathology scale are in the medium risk category. Again, there was only 1 interaction term statistically significant when analysing the CAPE sum score (PT*medium risk), while were more interaction terms were statistically significant when analysing CAPE positive symptoms (PT*medium risk and NTPT * medium and high risk).

### Main effect aberrant salience and psychotic experiences

Aberrant salience was hypothesised to be positively associated with psychotic experiences. If so, this type of experience could be interpreted as an early manifestation of psychotic symptoms*. *However, the present results indicated that there was no statistically significant association between the presence of aberrant salience and psychotic experiences, neither when analysing the psychotic symptom sum score nor when analysing positive symptoms. This is in line with the findings reported by Pries and colleagues [16]. These authors replicated the methods of two previous studies [13, 15] and established that there was no association between speech illusions and CAPE scores. Our results are in concordance with previous studies indicating no statistically significant association between psychotic experiences and speech illusion [1, 15, 16, 35]. On the other hand, the first study by Galdos and colleagues [13] showed a main effect in positive symptoms and although Catalan [15] did not find any statistically significant difference, there was a non-significant main effect in positive symptoms, only.

In addition, previous studies, using slightly different concepts, did show measures of aberrant salience to be associated with psychotic symptoms or psychotic experiences in daily life in ultra-high risk patients and in first episode psychotic disorder patients. In these studies, aberrant salience was either assessed using self-report or using a laboratory task (white noise test or salience attribution test) and schizotypy was assessed using interview measures or using multiple self-assessments on psychotic experiences per day [5, 6, 9–11, 13, 14].

### Moderation by genetic vulnerability

Modifiers in the association between aberrant salience and psychotic experiences (Fig. 1) could be the reason why Galdos and colleagues [13] reported an association between aberrant salience and psychotic experiences in healthy controls while attempts to replicate in general population samples failed [16]. Our results showed that PRS is a modifier in the analysis between ambiguous situations and positive symptoms. When analysing total psychotic symptoms evidence that PRS is a modifier was weaker. As far as we know, this is the first study analysing this interaction. The earlier shown differences in main effects when analysing the positive subscales make the interaction in positive symptoms plausible, but this needs to be replicated.

Whether PRS as a global summary measure is a modifier or not, specific genetic factors might be identified as the true modifiers. This is beyond the scope of the present analysis. Future research can study specific genetic make-up known to be involved in the aberrant salience mechanism.

### Explanation of conflicting results

Results of previous research are summarised in Table 4. After the first publication [13], cross-sectional general population studies analysing the association between aberrant salience and psychotic experiences mainly showed null-findings, but there were a few exceptions. The present results showed interaction between aberrant salience and genetic vulnerability in positive symptoms and a previous study showed a non-significant association between aberrant salience and positive symptoms [15]. When using the experience sampling method (ESM, i.e. assessments at multiple semi-random time point during various consecutive days), an association between aberrant salience and psychotic experiences was shown [14].

**Table 4:** Association between aberrant salience and psychosis, schizophrenia or psychotic symptoms in literature

	Sample sizes	Sample	***Results***^a, b^	Outcome: disorder or symptoms	Aberrant salience assessment
**General population studies**					
Galdos 2011 [13]	Patients: 30 Siblings: 28 GP controls: 307	Consecutive admissions at psychiatric hospital, siblings and GP controls	In controls, association between speech illusion and positive schizotypy	SIS-R^c^	White noise task, task
Catalan 2014 [15]	FEP: 54 GP controls: 150	GP controls and patients with FEP (see below)	In controls, **NO** association between speech illusions and positive symptoms	CAPE, SIS-R	White noise task, task
Pries 2017 [16]	Twins: 704	Sample of adolescent GP twins	**NO** association between speech illusions and psychotic experiences (CAPE: positive, negative or depressive; SIS-R: positive dimension, negative dimension)	CAPE positive, negative and depression sub scales, SIS-R	White noise task, task
Gonzalez de Artaza 2018 [1]	GP adults: 185	GP adults	After adjustment for confounders^d^ positive symptoms was **NOT** associated with dichotomised aberrant salience	SIS-R, CAPE	White noise task, task
Schepers 2019 [37]	GP adults: 112 (95 at follow-up)	GP adults	No association between aberrant salience and CAPE positive scale	Positive symptom subscale of the CAPE	White noise task
Chun 2020 [14]	Young adults: 165	Young adults recruited from undergraduate course, oversampled schizotypy	Aberrant salience was associated with psychotic-like experiences in daily life	ESM-data to assess schizotypy^h^	ASI, self-report
Current study	Twins: 887	Sample of twins (see Pries [16]), longitudinal data	**NO** association between aberrant salience and psychotic experiences	CAPE sum score and positive symptom subscale	White noise task, ambiguous situations task, tasks
**Ultra high risk and first episode of psychosis patient populations (cf GP controls)**					
Roiser 2009 [38]	FEP/schizophrenia: 20 GP controls: 17	Medicated (and 3 unmedicated) FEP and GP controls (advertisements), age 16–50	**NO** difference in aberrant salience between patients and controls. **However,** in patients, presence of delusions was associated with aberrant salience	Patients (DIP, DSM-IV) cf. controls	SAT (reaction time), task
An 2010 [9]	FEP: 20 UHR: 24 GP Controls: 39	UHR, FEP^e^ and GP controls	Both UHR and FEP have more attribution bias for perceiving hostility than controls	UHR, FEP, controls	AIHQ, self-report
Galdos 2011 [13]	Patients: 30 Siblings: 28 GP controls: 307	Consecutive admissions at psychiatric hospital, siblings and GP controls	Aberrant salience more prevalent in patients. Dose–response aberrant salience with increased familial risk	Psychotic disorder defined using DSM-IV	White noise task, task
Roiser 2013 [5]	UHR: 18 GP controls: 18	UHR individuals presenting at a clinical service for ARMS and GP controls	UHR score higher on an aberrant salience task than GP controls	UHR cf. controls (CAARMS)	SAT, task
Catalan 2014 [15]	FEP: 54 GP controls: 150	Convenience sample of FEP patients and GP controls	FEP patients had a higher rate of speech illusions (= aberrant salience)	FEP meeting DSM-IV-TR criteria cf. controls	White noise task, task
Smieskova 2015 [39]	FEP: 29 ARMS: 34 GP controls: 19	FEP, ARMS, GP controls	**NO** differences between the 3 groups	CAARMS, ICD-10 or DSM-IV	SAT, task
Reininghaus 2016 [40]	FEP: 51 ARMS: 46 GP controls: 53	FEP, ARMS, GP controls, aged 18–64 (ARMS 18–35)	Aberrant salience higher in ARMS and FEP than in controls	ARMS and FEP defined using ICD-10 and CAARMS or SPI-A respectively, vs controls	3 ESM-items^f^
Klippel 2017 [41]	FEP: 51 ARMS: 46 GP controls: 53	FEP, ARMS, GP controls	Aberrant salience higher in ARMS and FEP than in controls	ARMS and FEP defined using CAARMS and SCID vs controls	3 ESM-items^f^
Schmidt 2017 [6]	UHR: 23 GP controls: 13	UHR recruited from outreach and support in South London and GP controls	UHR score higher on aberrant salience task than controls	UHR cf. controls (CAARMS)	SAT, task
Pelizza 2021 [10]	FEP: 104 UHR: 45 CAARMS-: 55	Young community help seekers, aged 13–35 years. Including CAARMS-, UHR and FEP	FEP and UHR have more aberrant salience than CAARMS- individuals Correlation between ASI and positive symptoms scale of CAARMS showed concurrent validity in the total sample	UHR, FEP and CAARMS-, defined using CAARMS	ASI, self-report
Scazza 2021 [12]	FEP:139	Specialist help seeking, aged 13—35 years	FEP had high scores on aberrant salience, but FEP was the only group included in the study	FEP defined using CAARMS	ASI, self-report
Poletti 2022 [11]	CAARMS-: 91 UHR: 87 FEP: 139	Specialist hel*p-*seeking, aged 13–35 years	UHR and FEP subjects similar levels of aberrant salience, but higher than CAARMS- group	UHR, FEP, CAARMS- defined using CAARMS	ASI, self-report
Pugliese 2022 [42]	Outpatients: 102	Outpatients diagnosed with schizophrenia spectrum disorders	Higher ASI total scores for patients not in remission than patients in remission ASI total was associated with PANSS	PANSS criteria for remission; PANSS	ASI (with 5 subscales), self-report
**Studies using ESM for aberrant salience as well as outcome in patient populations (NOTE some studies are also presented above for the cross-sectional results)**					
Reininghaus 2016 [40]	FEP: 51 ARMS: 46 Control: 53	FEP, ARMS, GP controls Aged 18–64 years (ARMS aged 18–35 years)	Association between aberrant salience and psychotic experiences in all 3 groups Stronger association between aberrant salience and psychotic experiences in ARMS than in (medicated) FEP and controls	ESM-psychosis measure^f^	3 ESM-items^f^
Klippel 2017 [41]	FEP: 59 ARMS: 51 GP controls: 55	FEP, ARMS, GP controls	Multiple multilevel moderated mediation model: ↑ Aberrant salience—> more intense PE in all 3 groups Simple model: Pathway ↑ event related stress—> ↓ aberrant salience—> ↓ PE; in FEP but not in ARMS or controls	ESM-psychosis measure^f^	3 ESM-items^f^
So 2018 [43]	Inpatients: 16	Inpatients with a diagnosis of schizophrenia spectrum or other psychotic disorder and persecutory delusions	Association between aberrant salience and level of paranoia as well as increase in paranoia at t + 1 (between 5 min and 3 h after t)	Paranoia in ESM^g^	3 ESM-items^g^

*AIHQ *Ambiguous Intentions Hostility Questionnaire (self-report)

*ARMS* At-Risk Mental State

*ASI* Aberrant Salience Inventory (self-report). The ASI has 5 subscales; (feelings of increased significance, sense sharpening, impending understanding, heightened emotionality, and heightened cognition), but only Pugliese [42] assessed the individual subscales

*CAARMS* Comprehensive Assessment of At-Risk Mental State

*CAARMS-* Below threshold scores on the CAARMS (as opposed to UHR and FEP) in help-seeking populations

*CAPE* Community Assessment of Psychic Experiences

*DIP* Diagnostic Interview for Psychosis

*DSM*  Diagnostic Statistical Manual of Mental Disorders

*ESM* Experience Sampling Method (multiple assessments per day for several days)

*FEP* First Episode Psychosis patients

*GP* General Population

*OPCRIT* Operational Criteria Checklist for Psychotic Illness and Affective Illness

*PANSS* Positive and Negative Syndrome Scale

*SAT* Salience Attribution Test—> speeded response game rewarded with money

*SCID* Structured Clinical Interview for DSM Disorders

*SIS-R* Structured Interview for Schizotypy-Revised

*SPI-A* Schizophrenia Proneness Instrument—Adult version

*UHR* Ultra High Risk for psychosis individuals

^a^When one of the studies is longitudinal, the table presents the cross-sectional aberrant salience results

^b^The table reports results from positive symptoms; negative symptoms are not included in the table, usually no association

^c^age, sex and intelligence quotient

^d^In fact, the authors used the term first episode schizophrenic patients

^e^(a) Aberrant salience in ESM (1) “Everything grabs my attention right now” (2) “everything seems to have meaning right now” (3) “I notice things I haven’t noticed before”, 7 point Likert scale. Modified from [43–45].  (b) Psychosis measure in ESM “I feel paranoid,” “I feel unreal,” “I hear things that aren’t really there,” “I see things that aren’t really there,” “I can’t get these thoughts out of my head,” “My thoughts are influenced by others,” “It’s hard to express my thoughts in words,” and “I feel like I am losing control”; 7-point Likert scale [46, 47]

^f^(a) Aberrant salience in ESM (1) “At this moment, how much do things around you grab your attention?” (2) “At this moment, how much do you feel that everything seems to have some meaning?” (3) “At this moment, how much do you notice things that you have not noticed before?””, 7 point Likert scale.  (b) Psychosis measure in ESM “How suspicious do you feel right now?”, 7 point Likert scale

^g^“Right now my thoughts are strange or unusual”, “Right now my sight or hearing seems strange or unusual”, “Since the last signal, I have heard or seen things others could not”, “Right now I feel that someone or something is controlling my thoughts or actions”, “Right now familiar things seems strange or unusual”, 7 point Likert scale, assessed 8 times a day between 10 am and 10 pm

Despite the conflicting results in the general population, findings in ultra-high risk patients and in patients with first episode psychosis compared with general population controls were more robust, as illustrated in Table 4. There are various reasons for the contradictory findings in the general population and ultra-high risk and patient populations. First, the general population studies mainly used aberrant salience tasks (white noise task, reaction time task, Table 4), while the studies that did report an association mainly assessed aberrant salience using self-report. A reason for the contradictory findings could be that aberrant salience cannot be reliably assessed using self-report. However, not all studies in ultra-high risk and patients’ populations used self-report instruments and not all studies in the general population used tasks. Second, there could be a true difference in findings between general population subjects on the one hand and ultra-high risk and patient populations on the other. In that case, patient status (general population subject, ultra-high risk or patient) is a modifier (Fig. 1). It could be plausible that patient status is a modifier in the association between aberrant salience and positive symptoms. In patients and ultra-high risk subjects, dopamine signalling may be amplified as a consequence of inaccurate processing of stimuli, as discussed in the introduction [3, 4, 7]. In the prodromal phase, dopaminergic dysfunction may be involved in the coming into existence of aberrant salience [42]. It is likely that this appears not only in the prodromal phase, but also in ultra-high risk phases. This could explain why results in the general population are different from results in patient and ultra-high risk populations.

There is also evidence for an association between aberrant salience and psychotic experiences in general population subjects when both are assessed multiple times a day using ESM (Table 4 [40, 41]). ESM is a different level of measurement. Variation over the day within persons is only studied twice and replication is not possible using the present data. This finding as well as the possible conflict with the present conclusion needs to be assessed in future research.

## Conclusion

Although previous research showed evidence for an association between aberrant salience and psychotic symptoms in ultra-high risk and patient populations, evidence for a similar main effect in the general population is weak. The seemingly conflicting findings in the general population could be the result of modification by genetic background.

## Data Availability

The datasets analysed during the current study are not publicly available, but are available from the corresponding author on reasonable request. Reason is that data are from individuals; putting them online is against general privacy rules and GDPR.

## Abbreviations

CAPE: Community Assessment of Psychic Experience
EFPTS: East Flanders Twin Survey
ESM: Experience sampling method
GWAS: Genome-wide association study
NF: Negative foil
NT: Negative target
NTPT: Sum score of NT and PT
PRS: Polygenic risk score
PF: Positive foil
PT: Positive target
